# The anti-aging properties of a human placental hydrolysate combined with dieckol isolated from *Ecklonia cava*

**DOI:** 10.1186/s12906-015-0876-0

**Published:** 2015-10-05

**Authors:** Su Kil Jang, Do Ik Lee, Seung Tae Kim, Gwang Hoon Kim, Da Woon Park, Jung Youl Park, Daehee Han, Jae Kwon Choi, Yoon-bok Lee, Nam-Soo Han, Yun Bae Kim, Jeongsu Han, Seong Soo Joo

**Affiliations:** Department of Marine Molecular Biotechnology, College of Life Science, Gangneung-Wonju National University, 120 Gangneung Daehangno, Gangneung, Gangwon, 210-702 Republic of Korea; College of Pharmacy, Chung-Ang University, Heuksuk-dong, Dongjak-gu, Seoul, 156-756 Republic of Korea; Industry-Academic Cooperation Foundation, Hanbat National University, Daejeon, 305-719 Republic of Korea; Central Research Institute, Dr. Chung’s Food Co. Ltd., Chungbuk, 361-782 Republic of Korea; Chungbuk National University, Chungbuk, 361-763 Republic of Korea; DF-Dr. Han Biotech., Shaoyaojubeili, Chaoyang District, Beijing, 10029 China

**Keywords:** Human placental hydrolysate, Dieckol, Muscle, Cognition, Collagenase, Mitochondria

## Abstract

**Backgrounds:**

In the present study, we aimed to examine the anti-aging properties of human placental hydrolysate (HPE) and dieckol (DE) from *Ecklonia cava* against free radical scavenging, muscle hypertrophy-related follistatin mRNA expression, amelioration of cognition-related genes and proteins, inhibition of collagenase-regulating genes, and elastinase activity.

**Methods:**

The anti-aging effects were examined in human fibroblast (CCD986sk), mouse myoblast (C2C12), and neuroblastoma (N2a) cell models, by employing various assays such as 2,2-diphenyl-1-picrylhydrazyl hydrate (DPPH) scavenging, hydroxyl radical-mediated oxidation, quantitative real-time polymerase chain reaction, enzyme activity, and immunocytochemistry observation.

**Results:**

Our results show that HPE combined with DE (HPE:DE) strongly scavenged DPPH radicals and protected proteins against degradation by hydroxyl radical attack. HPE:DE effectively inhibited matrix metalloproteinase-1 expression, protein kinase C alpha expression, and elastinase activity. Furthermore, HPE:DE improved the expression of cognition-related genes (choline acetyltransferase and vesicular acetylcholine transporter). These events may proactively contribute to retard the aging processes and the abrupt physiological changes probably induced by mitochondrial dysfunction with aging.

**Conclusions:**

Based on these findings, we conclude that the combined treatment of HPE:DE may be useful for anti-aging therapy in which the accumulation of oxidative damage is the main driving force.

## Background

Aging is a series of biological changes that follow a natural progression from birth to death and is a multidimensional process of physical, psychological, and social changes. Identifying the major contributing factors to aging and increasing longevity without age-related illness is a cherished desire for human beings. Although much scientific knowledge has accumulated, preventing aging and prolonging lifespan continue to be a focus of attention. Aging-associated diseases that are not age-specific include atherosclerosis and cardiovascular disease, cancers, arthritis, cataracts, osteoporosis, type 2 diabetes, hypertension, and Alzheimer’s disease [1]. Excess production of free radicals may cause age-related impairment through oxidative damage to biomolecules, and mitochondria are the main target of free radical attack [2–4]. In addition, age-associated cognitive decline and neurogenic impairment, which may be caused by reduced superoxide dismutase and increased oxidative stress during aging, are important during aging but not fully understood [5, 6]. Human placenta, which includes diverse bioactive molecules, has attracted attention for managing the aging process [7, 8]. The placenta also possesses anti-oxidative, anti-inflammatory, anti-melanogenic, and collage-synthesizing properties that are effective anti-aging agents and rejuvenating to the body [9–11]. Dieckol (DE) was recently isolated from *Ecklonia* species, and this oligomeric polyphenol of phloroglucinols [12] has been reported to have diverse biological activities, such as antioxidant [13], anti-plasmin inhibitory [14], anti-mutagenic, anti-bacterial [15], anti-viral [16], tyrosinase inhibitory [17], anti-adipogenic [18], and matrix metalloproteinase-1 (MMP-1) inhibitory activities [19]. Thus, we hypothesized that increased free radical production may play a central role in aging and cause muscle and neuronal damage. In this study, we report the optimal effects of a human placental hydrolysate (HPE) combined with DE by focusing on the enhancement of aging-related indices, such as oxidative stress and muscle and cognitive impairment.

## Methods

### Sample preparation

Fresh *E. cava* was collected from the Jeju Island coast of South Korea in February 2013. A voucher specimen (NIBRAL0000145247) was authenticated by Prof. Joo (Biopharmaceutical Lab, College of Life Science, Gangneung National University, Republic of Korea), and deposited at the National Institute of Biological Resources, Incheon, Republic of Korea. Epiphytes, salt, and sand were completely removed with tap water. The samples were sanitized with 70 % ethanol, rinsed with deionized water, and freeze-dried. Finely ground *E. cava* (100 g) was steeped in 1 L of 80 % aqueous ethanol for 24 h repeatedly for 3 days at room temperature. The ethanol hydrolysates were combined, filtered through filter paper (Whatmann International Ltd., Maidstone, UK), evaporated, and dried completely. After the hydrolysate was suspended on 1 L distilled water, the organic soluble fraction was obtained with ethyl acetate. Finally, DE was obtained by purifying the polar fraction using the Prep-LC (LC-9104, JAI) system equipped with an ODS column in methanol solvent as described previously [20]. The HPE (Laennec, human placenta hydrolysate) was obtained from Japan Bioproducts Industry Co., Ltd. (Tokyo, Japan).

### Amino acid analysis

Amino acid concentrations were measured with an automatic amino acid analyzer (L-8800; Hitachi, Tokyo, Japan). Sample aliquots containing 8–12 mg protein were placed in a 20-mL cuvette and mixed with 9 mL of 6 M HCl. After sealing the cuvette, the samples were hydrolyzed at 110 °C for 24 h under N_2_. The hydrolysates were transferred to a 100 mL volumetric flask, mixed with 9 mL 6 M NaOH, and diluted with 0.02 N HCl. Then, all samples were filtered and loaded in a Hitachi L-8800 amino acid analyzer for the analysis.

### Radical scavenging and protein protection assays

The 2,2-diphenyl-1-picrylhydrazyl hydrate (DPPH) radical is one of the few stable organic nitrogen radicals and has a deep-purple color. Fractions were reacted with the DPPH solution to evaluate free radical scavenging activity. Each lyophilized fraction was dissolved in dimethyl sulfoxide (DMSO, Sigma-Aldrich) as a stock solution (100 mg/mL), and each fraction was reacted with 0.3 mM DPPH in methanol. Various concentrations of HPE or DE (0.01–100 μg/mL) were reacted with the DPPH radical solution for 20 min at room temperature, and absorbance was measured at 517 nm. DPPH free radical scavenging activity was calculated using the following equation: DPPH scavenging activity (%) = [Ac – (A – As)]/Ac × 100, where Ac is the absorbance of the control DPPH solution, A is absorbance of the sample with the DPPH solution, and As is absorbance of the sample. Hydroxyl radical-mediated oxidation experiments were performed for the protein protection assay using a metal-catalyzed reaction, as described previously with some modifications [21]. The target protein, bovine serum albumin (BSA), was dissolved in a 150 mM phosphate buffer (pH 7.3) to a final concentration of 0.5 mg/mL. The BSA solution was incubated with and without 100 μM copper (Cu^2+^) and 2.5 mM H_2_O_2_ in the presence and absence of the samples. The control antioxidant was 0.1 mM ascorbate, which was directly dissolved in PBS. The reactions were carried out in open tubes and placed in a shaking water bath maintained at 37 °C. After the reaction was complete, each mixture was separated by 10 % sodium dodecyl sulfate-polyacrylamide gel electrophoresis and stained with 0.1 % Coomassie Blue Brilliant solution.

### Elastase inhibition assay

This assay was performed in 0.2 mM Tris–HCl buffer (pH 8.0) in accordance with a previous study with minor modifications [22]. In brief, porcine pancreatic elastase (Sigma-Aldrich) was dissolved to prepare a 3.33 mg/mL stock solution in sterile water. The N-succinyl-Ala-Ala-Ala-*p*-nitroanilide substrate was dissolved in buffer to 1.6 mM. The test hydrolysates were incubated with the enzyme for 20 min before adding substrate to begin the reaction. The final reaction mixture (250 μL total volume) contained buffer, 0.8 mM substrate, 1 μg/mL enzyme, and various concentrations of HPE, DE, and HPE:DE, as indicated. Asc (100 μM) was used as the positive control. Absorbance values between 381 and 402 nm were measured immediately following addition of the substrate and then continuously for 20 min using a Spectra Max 340 Microplate Reader in Nunc 96 well microtiter plates. The percent inhibition of elastase was calculated as follows: Inhibition (%) = [(ODcontrol − ODsample)/ODcontrol] × 100.

### Cell culture

Human fibroblast (CCD986sk), mouse myoblast (C2C12), and neuroblastoma cell lines (N2a) (Korean Cell Line Bank, Seoul, Republic of Korea) were grown in Dulbecco’s Modified Eagle’s Medium (DMEM) (Hyclone. Logan, UT, USA.) supplemented with 10 % fetal bovine serum, 100 U/mL penicillin, and 100 μg/mL streptomycin (Invitrogen, Carlsbad, CA, USA.). The cultures were maintained under 5 % CO_2_ at 37 °C in tissue culture flasks. The cells were grown to > 90 % confluency and subjected to no more than 20 cell passages. Media were changed every 2–3 days. Subconfluent cells were harvested and seeded at a density of 5 × 10^5^ cells or 1.5 × 10^6^ cells into poly-L-lysine-coated 35-mm or 60-mm culture plates. After plating for 24 h, the medium was replaced with serum-free DMEM, washed once with phosphate-buffered saline (PBS), and treated with HPE, DE, or the positive controls of phorbol myristic acetate (PMA), and ascorbic acid (Asc) (Sigma-Aldrich, St. Louis, MO, USA.).

### Cell viability

Cell viability in response to HPE and DE stimulation was investigated in 96-well microtiter plates (2 × 10^4^ cells/mL) following a 24-h culture using the Cell Counting Kit-8 (CCK-8; Dojindo Laboratories, Kumamoto, Japan). This system uses WST-8 [2-(2-methoxy-4-nitrophenyl)-3-(4-nitrophenyl)-5-(2,4-disulfophenyl)-2H-tetrazolium, monosodium salt], which produces water-soluble colored formazan upon bioreduction in the presence of the electron carrier, 1-methoxy-5-methylphenazinium methylsulfate. The plates were measured at 450 nm (Spectra Max 340, Molecular Devices, Sunnyvale, CA, USA.), and data from triplicate cultures are expressed as percent viability vs. the control.

### Quantitative real-time polymerase chain reaction (PCR) assay

Total RNA hydrolysates from each cell line were prepared using the Trizol method (Invitrogen). cDNA was synthesized from RNA by reverse transcription of 1 μg of total RNA using the Improm-II reverse transcription system (Promega, Madison, WI, USA.) and oligo dT primers in a total volume of 20 μL. PCR amplification was performed using the primers described in Table 1 (Bioneer, Deajeon, Republic of Korea). Quantitative real-time PCR reactions were run on a Rotor-Gene 6000 (Corbett Research, Sydney, Australia) using SYBR Green PCR Master Mix (Qiagen, Valencia, CA, USA.) in 20-μL reaction mixtures. Each real-time-PCR master mix contained 10 μL 2× enzyme Mastermix, 7.0 μL RNase free water, 1 μL of each primer (10 pM each), and 1 μL diluted template. The PCR was performed with an initial pre-incubation step for 10 min at 95 °C, followed by 45 cycles of 95 °C for 15 s, annealing at 52 °C for 15 s, and extension at 72 °C for 10 s. A melting curve analysis was used to confirm formation of the expected PCR product, and products from all assays were tested additionally by 1.2 % agarose gel electrophoresis to confirm the correct lengths. An inter-run calibrator was used, and a standard curve was created for each gene to obtain PCR efficiencies. Relative sample expression levels were calculated using Rotor-Gene 6000 Series Software 1.7 and were expressed relative to glyceraldehyde 3-phosphate dehydrogenase and corrected for between-run variability. Data are expressed as a percentage of the internal control gene.

**Table 1:** Primer sequences used for the real-time polymerase chain reaction analysis

Gene		Primer	Amino acid sequence	Product size (bp)	Accession No.
Human	MMP1	5′ Primer	5′- TAGTGGCCCAGTGGTTGAAA	228	NM_002421
		3′ Primer	5′-CCAGATTTGCCAAGAGCAGA		
	PKCα	5′ Primer	5′-CCTTTCCTTTGGAGTTTCGG	228	NM_002737
		3′ Primer	5′-CCAACAACCTTGACCGAGTG		
	GAPDH	5′ Primer	5′- GGAGCCAAAAGGGTCATCAT	203	AK_026525
		3′ Primer	5′- GTGATGGCATGGACTGTGGT		
Mouse	MAP-2	5′ Primer	5′- ACCACACCTGCAGTGGAGAA	227	M21041
		3′ Primer	5′- AATCTGGACCTGGTTCCTGC		
	NGF	5′ Primer	5′- TACTGCACCAATAGCTGCCC	191	NM_013609
		3′ Primer	5′- TTTCAACAGGACTCACCGGA-		
	FSTN	5′ Primer	5′- GCTCTCTCTGCGATGAGCTG	174	NM_008046
		3′ Primer	5′ ATCTCGGAAGAAACGGAGGA-		
	β-actin	5′ Primer	5′-TACAGCTTCACCACCACAGC	187	NM_007393
		3′ Primer	5′-AAGGAAGGCTGGAAAAGAGC		

### Immunocytochemistry (ICC) and microscopic observations

Cultured N2a cells were fixed in 4 % paraformaldehyde in PBS for 15 min, washed twice with PBS supplemented with 100 mM glycine for 5 min, and incubated with permeabilization buffer consisting of 0.1 % Triton X-100 (Sigma-Aldrich) in PBS for 30 min at room temperature. Blocking was performed with 1 % BSA for 30 min at room temperature as previously described [23]. Then, choline acetyltransferase (ChAT) or vesicular acetylcholine transporter (VAChT) mouse monoclonal antibody (1:200; Santa Cruz Biotechnology, Santa Cruz, CA, USA.) was added to 1 % BSA in PBS with Tween 20 and incubated for 2 h at room temperature. The cells were washed three times with PBS before fluorescein isothiocyanate-conjugated anti-mouse immunoglobulin G (1:200; Cell Signaling Technology, Danvers, MA, USA.) was added to 1 % BSA for 1 h at room temperature. The cells were rinsed and counterstained with 4,6-diamidino-2-phenylindole (Sigma-Aldrich) for 10 min, followed by two PBS washes. The cultures were visualized with an inverted fluorescent microscope system (Eclipse Ti-S; Nikon, Tokyo, Japan) at a magnification of × 600.

### Statistical analysis

Statistical comparisons between groups were performed using one-way analysis of variance with Dunnet’s post-hoc test and SPSS v. 17 software (SPSS, Inc., Chicago, IL, USA.). A *p* < 0.05 was considered significant.

## Results and discussion

Among many the age-related changes that begins in adulthood, muscle weakness, cognitive decline, and the accumulation of reactive oxygen species (ROS) are closely related because ROS are major causative factors of aging through their oxidative deteriorating effects [24, 25]. Neurodegenerative diseases and the degenerative loss of skeletal muscle mass (sarcopenia) during aging are critically linked to mitochondrial dysfunction, which cannot functionally regulate or scavenge ROS via antioxidant enzymes, such as superoxide dismutase (SOD), catalase, and glutathione peroxidase [26, 27]. In addition, the main amino acid reservoir in the body is skeletal muscle, which contains approximately 75 % of all protein and progressively loses muscle mass and function during aging [28].

**Fig. 1:**
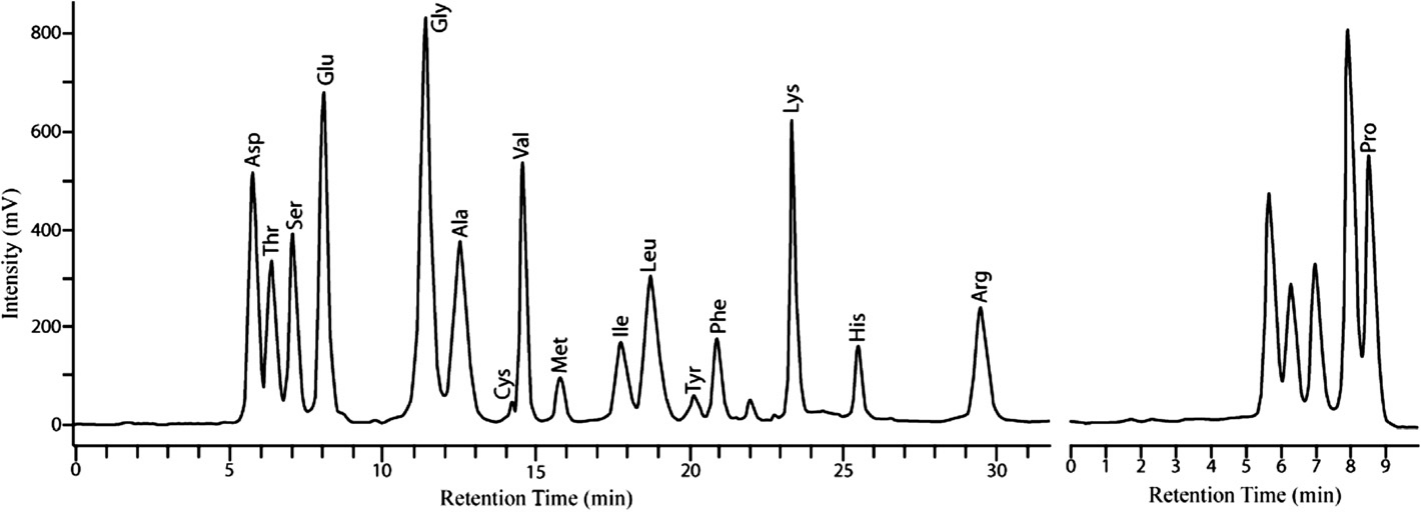
Typical amino acid chromatogram from the human placental hydrolysate (HPE)

In this respect, our results show that the HPE contained 17 amino acids, including nine essential amino acids and eight nonessential amino acids. Among the total amino acids, the quantity of sulfur-containing amino acids (cysteine and methionine) and aromatic amino acids (phenylalanine and tyrosine) was 0.62 and 1.82 g/100 g, respectively. As cysteine has potent anti-oxidant capacity, it is believed that the HPE may contribute to improve various age-related degenerative processes caused by ROS [29, 30]. Furthermore, the balanced essential and nonessential amino acids in the HPE may prevent the decline in baseline muscle protein synthesis, which promotes sarcopenia [31]. The amino acid profile of the HPE is shown in Table 2. Among the 17 amino acids, the major amino acids were glutamine (4.13 g/100 g), followed by glycine, asparagine, leucine, proline, lysine, arginine, alanine, and valine, which constituted > 76.3 % of the total amino acids contained in the HPE proteins (Table 2). The amount of nonessential amino acids was higher (52.9 %) than that of the essential amino acids (47.1 %). Figure 1 shows the amino acid chromatograms in the HPE.

**Table 2:** Amino acid composition

Classification	Amino acid	g/100 g	%
Sulphuric amino acids	Cysteine	0.13	0.4
	Methionine	0.59	1.7
Aromatic amino acids	Phenylalanine	1.32	3.8
	Tyrosine	0.49	1.4
Essential amino acids	Leucine	3.01	8.7
	Lysine	2.69	7.8
	Arginine^a^	2.68	7.7
	Valine	2.04	5.9
	Threonine	1.68	4.9
	Isoleucine	1.37	4.0
	Phenylalanine	1.32	3.8
	Histidine^a^	0.91	2.6
	Methionine	0.59	1.7
	(Sub-total)	16.29	47.1
Nonessential amino acids	Glutamine	4.13	11.9
	Glycine	3.5	10.1
	Asparagine	3.04	8.8
	Proline	2.74	7.9
	Alanine	2.58	7.5
	Serin	1.71	4.9
	Tyrosine	0.49	1.4
	Cysteine	0.13	0.4
	(Sub-total)	18.32	52.9

^a^Arginine and histidine form the so-called semi-essential amino acids

**Fig. 2:**
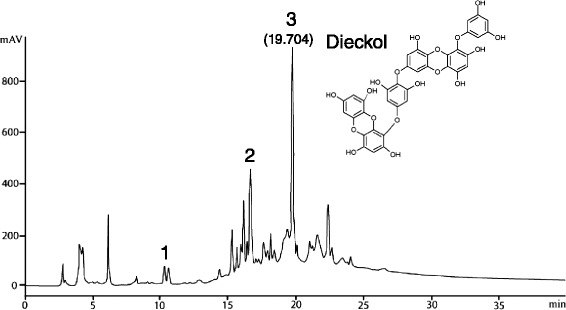
HPLC analysis of *E. cava* hydrolysate. Column: 4.6 mm × 250 mm. Separation was performed with a gradient from 5 to 60 % acetonitrile in 30 min at a flow rate of 1.0 mL/min. Elution was monitored at 230 nm (injection volume, 20 μL (1 mg/mL)). 1; phologlucinol, 2; eckol, 3; dieckol

In addition, we determined phlorotannins in a 70 % ethanol extract from *E. cava* using high performance liquid chromatography (HPLC) analysis (Fig. 2). Phlorotannins (phloroglucinol, eckol, and dieckol) was confirmed by comparing their liquid chromatography-mass spectrometry (LC-MS), proton Nuclear Magnetic Resonance (^1^H NMR) data to the previous report [13].

**Fig. 3:**
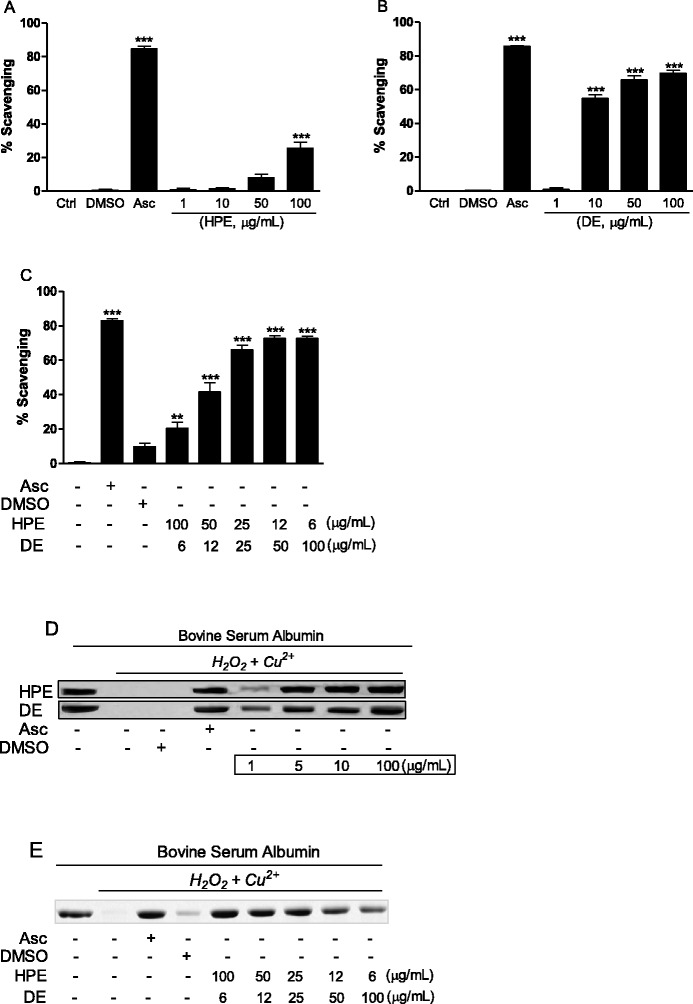
Radical scavenging activity. **a**-**c** DPPH free radical scavenging activity of the HPE, DE, and HPE:DE at different concentrations (1–100 μg/mL) was determined for a fixed time (20 min). **d**-**e** Polyacrylamide gel electrophoresis (PAGE) profiles show the protein obtained without treatment, with Cu^2+^/H_2_O_2_, and at different concentrations of the HPE or DE. Ascorbic acid (Asc. 0.1 mM) and 10 % DMSO were used as positive and vehicle controls, respectively. The final steps included incubating all of the reactants, including BSA, for 2 h, followed by 10 % sodium dodecyl sulfate-PAGE. Results are expressed as means ± standard deviations from three separate experiments. *P < 0.05, ***P < 0.001 vs. Ctrl. Ctrl, control

In the DPPH assay, HPE scavenged free radicals beginning at a concentration of 50 μg/mL, whereas DE showed higher activity at a lower concentration (10 μg/mL) (Fig. 3a, b). More enhanced scavenging effects were found when the two agents were combined (Fig. 3c). Notably, the combination of HPE (25 μg/mL) and DE (25 μg/mL) was the most beneficial concentration. This result was confirmed in the hydroxyl radical-mediated oxidation assay, which determined the protection of protein degradation. Degradation of BSA by hydroxyl radicals produced from Cu^2+^ and H_2_O_2_ was monitored in the presence of single HPE/DE or HPE:DE combination. As shown in Fig. 3d-e, hydroxyl radical scavenging activity was dose-dependently detected in both single treatments, whereas 25 μg/mL HPE:DE combination displayed high antioxidant activity. It is uncertain why higher DE and lower HPE combination displayed weak activity in protecting protein from hydroxyl radical attack. However, one possibility is that amino acids can act as a chelating agent for copper ions, thus alleviating generation of hydroxyl radical, while the multifunctional antioxidant activity of polyphenols is largely related to phenol rings which act as electron traps [32]. These scavenging effects indicate that the HPE:DE combination would provide more therapeutic advantages as an anti-aging therapy than those of a single component treatment.

**Fig. 4:**
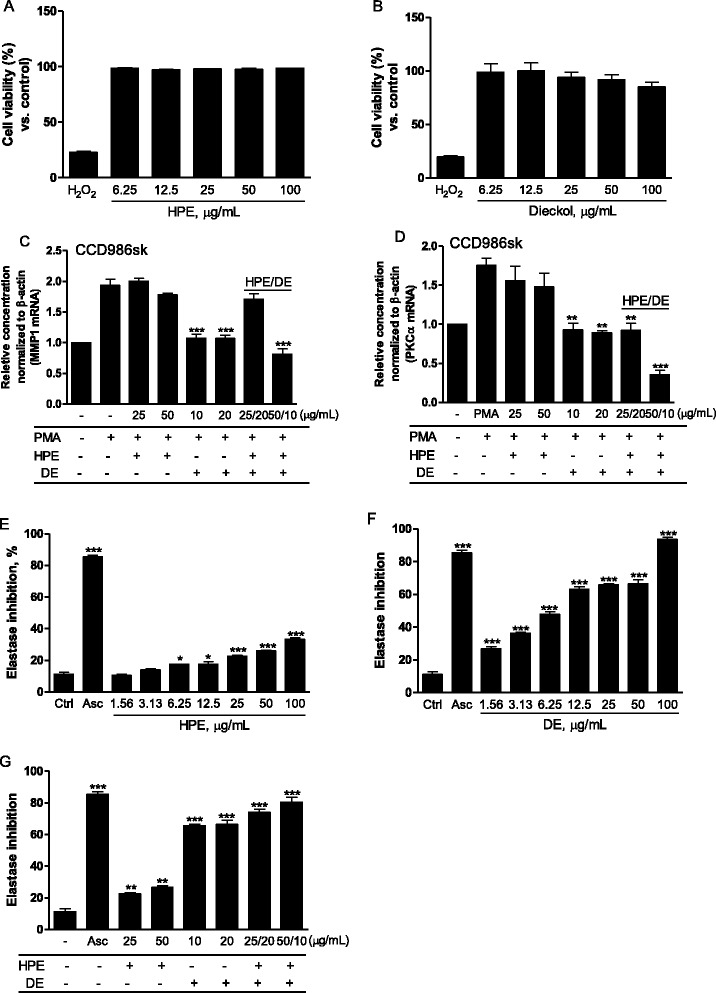
Effect on matrix metalloproteinase-1/protein kinase-α (MMP1/PCKα) gene expression in CCD986sk and elastinase activity. **a**, **b** Cell viability assays were performed, and the results were expressed as the percent viability for identical treatments of HPE and DE (6.25–100 μg/mL). Cells were seeded on 12-well culture plates and treated with the HPE and DE in the presence or absence of 50 μM phorbol myristic acetate (PMA) for 24 h. **c**, **d** MMP1 and PKCα mRNAs were quantified by fold units using the real-time polymerase chain reaction. **e**-**g** Elastase activity was measured between 381 and 402 nm immediately after adding the substrate. Results are expressed as means ± standard deviations from three separate experiments. *P < 0.05, **P < 0.01, ***P < 0.001 vs. PMA (**c** and **d**) or vs. Ctrl (**e**, **f**, and **g**)

MMP1 and PKCα mRNAs, which increase age-dependently, were examined in the CCD986sk human fibroblast cell line, which was not cytotoxic when incubated with HPE or DE at about 100 μg/mL. As collagen and elastin fiber atrophy in skin is predominant during aging due to increased expression of their degradative enzymes, the decrease of MMP1/PKCα mRNA expression would be the first choice for an anti-aging therapy. The results revealed that DE successfully inhibited MMP1 and PKCα mRNA expression, whereas HPE did not. However, both genes were remarkably inhibited at a higher concentration when the two were combined (50:10 μg/mL HPE:DE) (Fig. 4a and b), suggesting that HPE:DE results in efficient formation of collagen [33, 34]. Consistently, elastase activity was well inhibited after the DE and HPE treatments. Interestingly, optimal inhibition of elastase occurred after the combined HPE:DE treatment (Fig. 4c-e). These data strongly indicate that degradation of collagen and elastin fibers was diminished following the HPE:DE treatment. This indicates that HPE would synergistically play a role in skin revitalization and rejuvenation by improving skin elasticity and thickness along with enhancing skin texture [8].

**Fig. 5:**
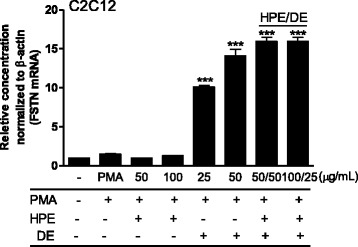
Effect on follistatin (FSTN) gene expression in C2C12. Cells were seeded on 12-well culture plates and treated with the HPE and DE in the presence or absence of 50 μM phorbol myristic acetate (PMA) for 24 h. FSTN mRNA was quantified by fold units using the real-time polymerase chain reaction. Results are expressed as means ± standard deviations from three separate experiments. ***P < 0.001 vs. PMA

Because muscle weakness and loss of muscle mass in the form of sarcopenia are major changes during aging, the increased protein synthesis and decreased protein degradation in hypertrophied muscle are important events in aging. Therefore, the overexpression of FSTN, which is essential for muscle fiber formation and growth, may be the major event regulating musculoskeletal aging [35]. Our data showed that HPE alone did not increase FSTN expression in C2C12 myoblast cells, whereas DE significantly increased FSTN expression, suggesting improved muscle fiber formation and growth. However, FSTN expression was much more enhanced following co-treatment with HPE:DE (Fig. 5). As the older muscle is still able to respond to amino acids, which have been shown to acutely stimulate muscle protein synthesis in older individuals, plenty amounts of leucine and glutamine in HPE are synergistically to stimulate muscle protein synthesis and to maintain muscle tissue by preserving lean tissue mass [36, 37].

**Fig. 6:**
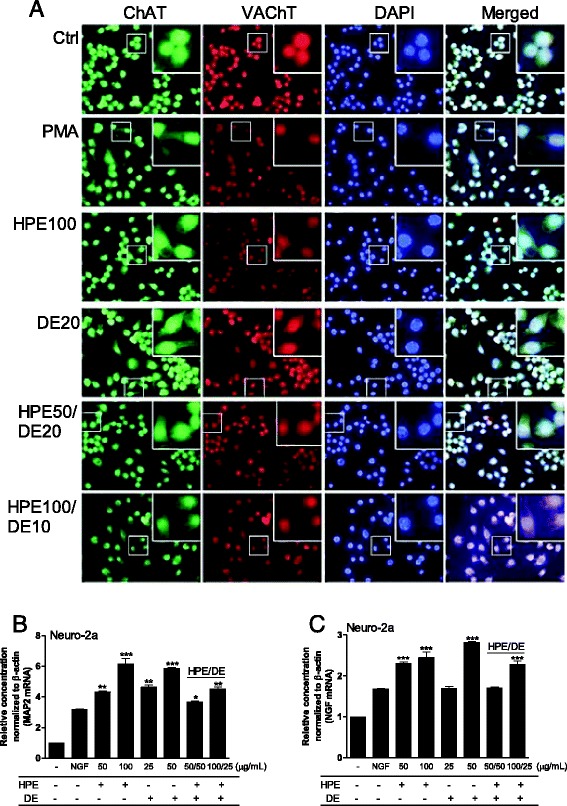
Immunostaining for choline acetyltransferase (ChAT)/vesicular acetylcholine transporter (VAChT) and MAP2/nerve growth factor (NGF) gene expression in Neuro2a (N2a) cells. **a** ICC shows that two major cholinergic markers, ChAT and VAhT were well expressed compared to those in the untreated control group. **b**-**c** Expression of MAP-2, a neuronal differentiation marker and NGF mRNAs, was quantified by fold units using the real-time polymerase chain reaction. ***P < 0.001 vs. NGF

On the other hand, we previously reported that ChAT overexpressing human neural stem cells restore cognition by increasing of acetylcholine levels in a rat model [38]. Thus, we evaluated whether HPE and DE increase ChAT and VAChT expression, which are required for cholinergic neurotransmission and coordinately contribute significantly to increase intracellular acetylcholine in cholinergic neurons [39]. ChAT and VAChT mRNA were distinctively expressed in N2a neuroblastoma cells, after the HPE and DE treatments (Fig. 6a), suggesting a functional contribution by HPE and DE in neuronal differentiation and cholinergic gene expression. Notably, MAP-2, a neuronal differentiation marker, and NGF mRNAs increased significantly either with HPE or DE alone or in combination, dose-dependently (Fig. 6b and c). Our data clearly showed that the HPE and DE effectively enhanced ChAT and VAChT expression and the significant increase in MAP-2 and NGF mRNA expression in N2a cells. These evidences clearly supported that either HPE:DE combination or single treatment can promote the differentiation and stable growth of neuronal cells, indicating an effective decrease against aging-induced cognitive impairments [40].

## Conclusions

The HPE:DE combination effectively improved free radical scavenging, muscle hypertrophy-related FSTN mRNA expression, ameliorated cognition-related genes (ChAT and VAChT) and proteins, and inhibited MMP1/PKCα expression and elastinase activity, suggesting that the combined treatment of HPE:DE may be useful for anti-aging therapy in which the accumulation of oxidative damage is the main driving force.
